# Supplementary material for: LiNi0.6Co0.2Mn0.2O2 Cathode-Solid Electrolyte Interfacial Behavior Characterization Using Novel Method Adopting Microcavity Electrode
Source: Molecules. 2023 Apr 17;28(8):3537. doi: 10.3390/molecules28083537 (PMC10144035; doi:10.3390/molecules28083537)
Supplement: Supplementary file 1 [file molecules-28-03537-s001.zip › molecules-2309912-supplementary.pdf]

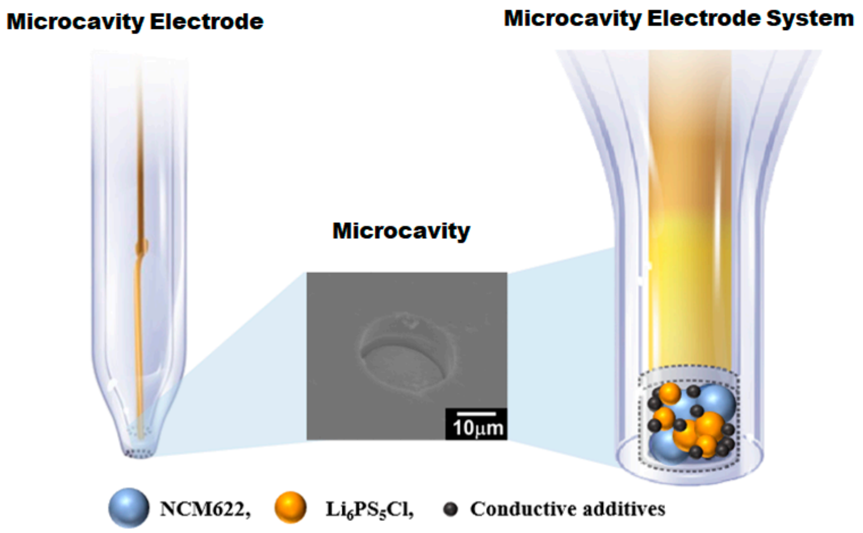

Figure S1. Pictorial representation of microcavity electrode, microcavity electrode system and image of the microcavity.

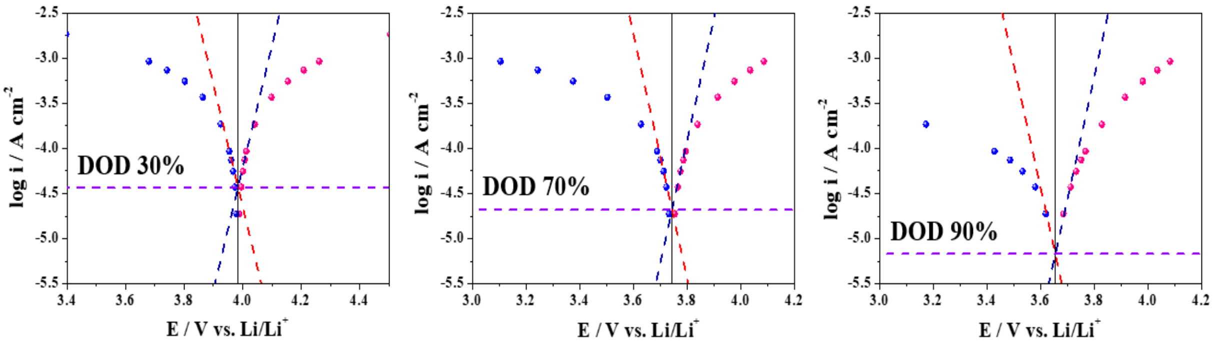

Figure S2. Tafel plots of NCM622- Li_6PS_5Cl system at DOD 30%, DOD 70%, and DOD 90% states.

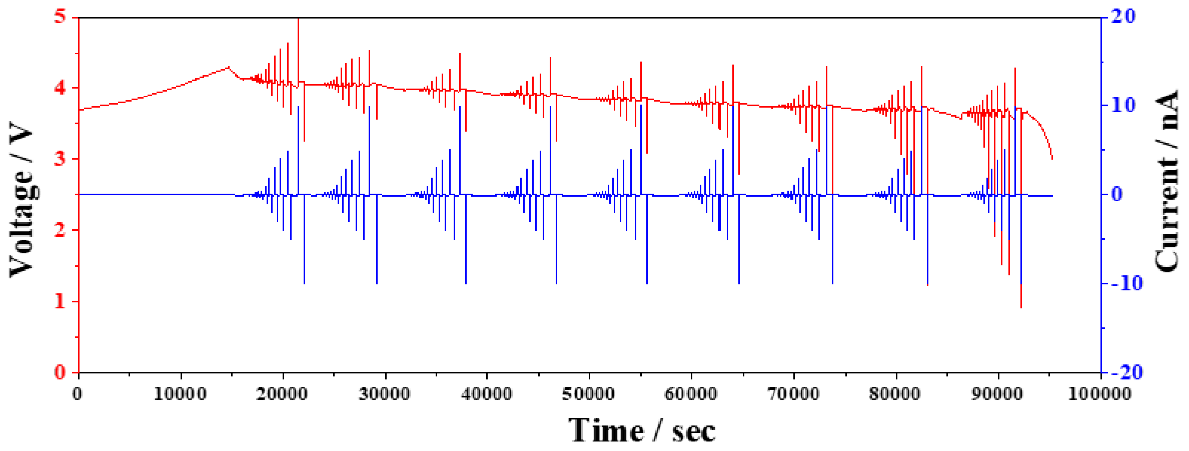

Figure S3. The applied pulse of various current densities in each DOD state to NCM622-Li_6PS_5Cl system.

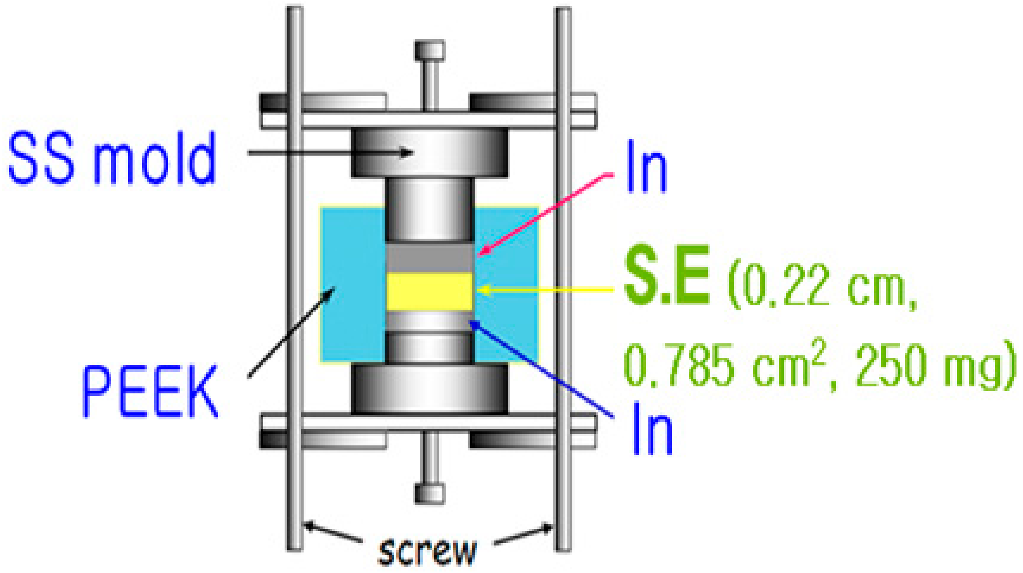

Figure S4. Pictorial representation of assembled pressure cell.

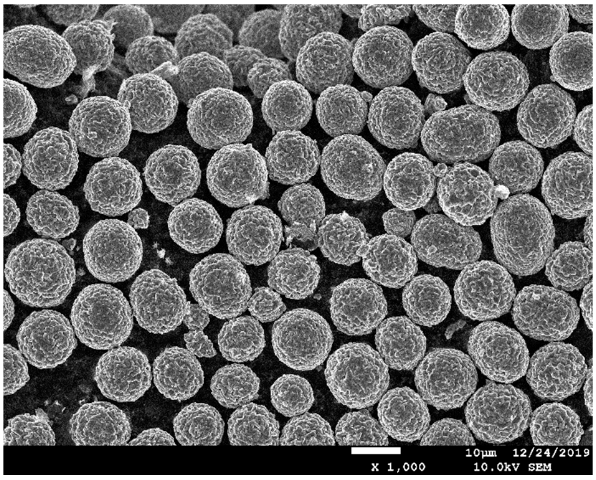

Figure S5. FE-SEM image of NCM622 cathode active materials.
